# Children of female sex workers and drug users: a review of vulnerability, resilience and family-centred models of care

**DOI:** 10.1186/1758-2652-13-S2-S6

**Published:** 2010-06-23

**Authors:** Jennifer Beard, Godfrey Biemba, Mohamad I Brooks, Jill Costello, Mark Ommerborn, Megan Bresnahan, David Flynn, Jonathon L Simon

**Affiliations:** 1 Center for Global Health and Development, Boston University, 801 Massachusetts Avenue, Boston, Massachusetts, USA; 2 Alumni Medical Library, Boston University Medical Center, 72 East Concord Street, Boston, Massachusetts, USA

## Abstract

**Background:**

Injection drug users and female sex workers are two of the populations most at risk for becoming infected with HIV in countries with concentrated epidemics. Many of the adults who fall into these categories are also parents, but little is known about the vulnerabilities faced by their children, their children's sources of resilience, or programmes providing services to these often fragile families. This review synthesizes evidence from disparate sources describing the vulnerabilities and resilience of the children of female sex workers and drug users, and documents some models of care that have been put in place to assist them.

**Review:**

A large literature assessing the vulnerability and resilience of children of drug users and alcoholics in developed countries was found. Research on the situation of the children of sex workers is extremely limited. Children of drug users and sex workers can face unique risks, stigma and discrimination, but both child vulnerability and resilience are associated in the drug use literature with the physical and mental health of parents and family context. Family-centred interventions have been implemented in low- and middle-income contexts, but they tend to be small, piecemeal and struggling to meet demand; they are poorly documented, and most have not been formally evaluated. We present preliminary descriptive data from an organization working with pregnant and new mothers who are drug users in Ukraine and from an organization providing services to sex workers and their families in Zambia.

**Conclusions:**

Because parents' drug use or sex work is often illegal and hidden, identifying their children can be difficult and may increase children's vulnerability and marginalization. Researchers and service providers, therefore, need to proceed with caution when attempting to reach these populations, but documentation and evaluation of current programmes should be prioritized.

## Background

Female sex workers (FSWs) and injection drug users (IDUs) are often categorized as two of the four populations "most at risk" for becoming infected with HIV due to behaviours that heighten their vulnerability to the virus. According to the Joint United Nations Programme on HIV/AIDS (UNAIDS), the term, "most-at-risk populations" (MARP), refers to men who have sex with men, injection drug users, sex workers and their clients.

These risk behaviours are believed to drive the HIV epidemics in western countries, former Soviet republics and Asia, where HIV is concentrated in specific populations [1].

Interventions for MARP tend to focus on the needs of adults, with the objective of reducing their risk for HIV through prevention, behaviour-change education and risk-reduction strategies. But, to date, little attention has been paid in the published literature to the vulnerabilities faced by their children or to interventions focused on keeping these potentially vulnerable families together, improving the wellbeing of both parents and children, and reducing the risk of both generations for becoming infected with or transmitting HIV.

This review aims to synthesize evidence from disparate sources (including research, advocacy and programmatic information) describing the vulnerabilities and sources of resilience of the children of female sex workers and drug users, and to document the two selected models of care in low-and middle-income countries that have been put in place to assist these groups. In the following sections, we analyze peer-reviewed and grey literature to begin to answer four research questions:

1. What are the vulnerabilities faced by the children of drug users and FSWs?

2. What are their sources of resilience?

3. Are there interventions that have focused on mitigating the vulnerability of children and addressing the needs of these families?

4. What do we know about the effectiveness or impact of these interventions?

While our original objective for this literature review was to focus specifically on the children of female sex workers and injection drug users in low- and middle-income countries, we found very little information specific to the children of IDUs. However, we did find a great deal of published work more broadly focused on drug and alcohol addiction in general. Likewise, we found that the most relevant literature on the children of drug users is from developed countries, and the United States in particular. As a result, we broadened our original scope in order to draw inferences from the global literature about the children of drug users of any type in low- and middle-income countries. By contrast, the literature on children of sex workers globally is limited, but the majority of the information we did find is focused on lower resource countries.

Synthesizing what is known about the types of vulnerability and resilience experienced by children of these groups, the types of assistance families need to minimize children's vulnerability, and the effectiveness of the interventions that exist is useful for several reasons. First, attention needs to be drawn to the reality that sex workers and drug users are often parents whose children potentially face vulnerabilities unique to their family situation. Second, understanding the needs of these children is necessary for creating relevant, evidence-based interventions focused on supporting their families. Finally, documenting the types of care that do exist and assessing their effectiveness is critical for scaling up and adapting successful interventions to new contexts.

### Literature review methodology

This literature review utilized both electronic and manual search methods to locate relevant peer-reviewed articles and grey literature from all low- and middle-income countries. We expanded our inclusion criteria to all countries regardless of income level only after our search for sources from lower resource contexts turned up little useful information. The following online databases and search engines were searched to identify relevant studies:Ovid/Medline, PubMed, Child Development and Adolescent Studies, PsychInfo, Published International Literature on Traumatic Stress, Sociological Abstracts, Social Services Abstracts, Web of Science, Google Scholar, Popline/One Source, the New York Academy of Medicine Grey Literature Report, and Public Affairs Information Service Archive. Organizational websites and references of all relevant sources were searched manually.

Our search paired the terms "parent", "child", "youth", and "orphan" with the following, using various combinations: "most-at-risk populations", "risk factors", "vulnerability", "resilience", "HIV/AIDS", "commercial sex worker", "female sex worker", "prostitution", "drug user", "drug use", "substance abuse", "substance abusing parents", "addiction", "intervention", "child care", "education", "prevention", "child victims", "injection drug use", "child welfare", "parent-child", and "child of impaired parents".

We also contacted staff from relevant programmes to ask about interventions being implemented for children of sex workers and drug users. Correspondence and phone interviews with these key informants provided the most relevant information on interventions in low- and middle-income countries.

### Terminology and definitions

This review faced a number of semantic challenges. First, the definition of "sex work" is profoundly unclear and runs a wide gamut of very different types of transactional sex, including but not limited to: brothel-based prostitutes; waitresses or bar girls who sell sexual favours within the establishments where they are employed; street walkers; dancing girls; caste-based *devadasis *in India; *kanjar *families in Pakistan; and courtesans or in *taiwaifs *South Asia who entertain men they call "husbands" and receive cash and other material gifts.

We looked at the children of sex workers who sometimes run the risk of entering the profession or being trafficked, but not at children who have been trafficked or who have entered prostitution through means other than "inheriting" it from their mothers. Nor did we examine the relationship between the children of FSWs and their fathers, who are often their mothers' clients (i.e., the fourth MARP category). For simplicity, we use the term, "female sex worker", to include all categories of women participating in transactional sex. We did not find information on the children of male sex workers.

As mentioned, the focus of this paper was shifted from the children of IDUs specifically to the children of drug users more generally to encompass the *drug use *or *substance abuse *literature, which includes research on the impact of all forms of parental drug use (including alcoholism) on children. While drug injection is specified in the literature on populations most at risk for HIV, we opted to include the more general drug use literature to inform our discussion of child vulnerability and resilience. But the intervention we describe later is specific to mothers who inject drugs.

Finally, we set out to look at the vulnerabilities and resilience of, and interventions for the children of drug users and sex workers, using search terms specific to each group. However, overlap between these two groups is common as drug use can create a gateway into sex work and vice versa [2-4]. We present information that is either generalizable across the two groups or distinct to each; however, we were not able to find data assessing the impact of "co-morbidity" on children whose parents are both drug users and sex workers.

### Estimating the number of drug users and female sex workers who are parents

Estimating the number of people within most-at-risk populations who are parents is extremely difficult. Drug users and sex workers are often parents, although this fact has generally been ignored in the MARP literature.

As noted by the UNAIDS Reference Group for Estimates, Modelling and Projections, "estimating the numbers and associated prevalence for high risk populations is a fundamentally difficult exercise" [5], creating a gap that undermines the validity of national estimates of HIV prevalence in concentrated epidemics [6]. For instance, while the United Nations Office on Drugs and Crime estimates 18-38 million "problem drug users" and 11-25 million injection drug users worldwide [7], we could not find global estimates of the proportion of drug users who have children.

Some country-specific estimates of children living with drug users have been calculated based on national household data. For instance, almost half a million children in the United Kingdom live with parents who reported drug use and problem drinking in the past year [8]. Not surprisingly, similar estimates of the number of children affected by parental drug use are not available for countries without similarly sophisticated, national healthcare tracking systems.

Overall global estimates of the number of female sex workers also could not be found. A global estimate of 40 million is sometimes cited by activists, but we were unable to find the source of that estimate. Vandepitte *et al *provide prevalence estimates of sex workers in urban areas of sub-Saharan Africa (0.7%-4.3% of the population), Asia (0.2%-2.6%), former Soviet countries (0.1%-1.5%), eastern Europe(0.4%-1.4%), western Europe (0.1%-1.4%) and Latin America (0.2%-7.4%) [9]. But they admit that their method of arriving at these estimates is precarious at best (and most likely conservative) due to inconsistent definitions of what sex work entails.

Likewise, global estimates of the number of sex workers who have children or of the number of children whose mothers are sex workers could not be found. Total fertility rates of sex workers globally have also not been documented in the searchable literature. While HIV and other sexually transmitted infections can reduce female fertility [6], the increased frequency of coital acts among sex workers also increases their exposure to pregnancy, arguably rendering their fertility to be little different from that of the general population. A study from Kenya reported that the mean number of children per their 385 sex worker respondents was 3.4 (± 2), making them comparable to the national mean of 3.2 [2].

Vietnam was the only country found to specify children of sex workers and drug users as vulnerable, along with children who have been trafficked, street children, and children who are themselves engaged in drug use and sex work. While the Ministry of Labour, Invalids and Social Affairs (MOLISA) is able to give estimates of the numbers of children who fall into the latter categories, it indicates that data is not available on children of sex workers or drug users [10].

### Sources of vulnerability and resilience for children of drug users and sex workers

The children of drug users and sex workers can face unique risks, stigma and discrimination as a result of their parents' addictions or profession. However, this potential vulnerability can be ameliorated by potential sources of resilience connected to support networks, parent health, parent-child bonding, education, economic situation and other environmental factors [11]. Research on the children of drug users in general focuses on their vulnerability to numerous forms of deprivation and abuse. A review of key articles from the past two decades yields a relatively long list of possible negative outcomes for children, ranging from cognitive developmental delays to neglect and abuse as a result of prenatal and postnatal exposure to parental addiction. However, research findings on the determinants of these various risks tend to be inconclusive, with family and community support networks, parental physical and mental health, and other socio-economic and environmental factors mediating child development outcomes and resilience [11-23].

The primary limitation of these research findings on possible vulnerabilities faced by children of drug users is that they come almost solely from high-income countries. Arguably, the risks and sources of resilience faced by children of addicted parents are potentially similar in contexts where certain drugs are illegal, drug use is stigmatized, and rehabilitation and risk-reduction programmes are difficult to access, if available at all. Overall, though, the generalizability of the information to low- and middle-income countries is unknown. At best, these findings can be useful for establishing research questions to be answered in lower resource contexts.

The literature on the children of sex workers, by contrast, is very small and, with a few exceptions, largely qualitative and ethnographic. While some useful articles look at US-based sex worker populations, most of the relevant research focuses on south Asia and Kenya. Specific vulnerabilities documented as affecting children of sex workers include: separation from parents, sexual abuse, early sexual debut, introduction to sex work asadolescents, low school enrolment, psychosocial issues arising from witnessing their mothers' sexual interactions with clients, and social marginalization [2,3,24-29]. The research on sex workers and their families tends to have a particular focus on girls and their potential for sexual abuse, early sexual debut, witnessing adult sexual activity, grooming to enter the trade, and trafficking. Sex work is often handed on from parent to child as the family trade in some cases, or out of a real or perceived lack of other options [28,30].

Sources of potential resilience for children of sex workers are also dependent on a complex combination of economic, environmental and social factors. Pardeshi and Bhattacharya found that *devadasis *had strong family support in their native villages [27]. While many of these women sent their children to their village homes to live with extended family, they remained connected with their children and visited at least once a year. Women who kept their children with them reported their income, peers, and brothels organized around native villages as sources of support. In Kenya, the more educated a sex worker was, the more likely she was to prioritize education for her children [2].

### Examples of family-centred interventions

Some interventions have been implemented in low- and middle-income countries to assist families of drug users and sex workers, but they tend to be small, piecemeal and struggling to meet demand. The few interventions directed at children of FSWs and drug users that we did find all started with a focus on adults, but expanded their services as parents sought care for their children. Family Health International, for instance, started providing health care to children of at-risk parents in Cote d'Ivoire as more parents started seeking care. Many of these parents had previously been unable to access support because their children do not fit the national definition of an orphan or vulnerable child.

### MAMA+ for IDU

Most information about family-centred care models for children of drug users comes from developed countries [12-14,16,31,32]. As Zuckerman notes, an addicted mother's interest in her baby is often the "healthiest" part of her life. But this interest is a double-edged sword that can exacerbate feelings of failure as much as provide a positive impetus to begin methadone maintenance or enter a rehabilitation programme [13,33].

In the US, drug rehabilitation programmes traditionally focused on the needs of men and did not accommodate a mother's reluctance to leave her children in order to enter residential treatment programmes. This started to change in the United States in the 1990s with the development of outpatient, family-focused treatment integrating screening of mothers during pregnancy for addiction and drug rehabilitation counselling, with, for example, primary health care for mothers and their children, legal assistance, food assistance and housing [13].

The MAMA+ for IDU project in Ukraine is the single programme outside of developed western countries for which we were able to find solid, if limited, information on provision of services to children or families of IDUs. As can be seen in Table 1, the integrated, family-centred, "one-stop shopping" model of care offered by MAMA+ is similar to that pioneered in the United States by Zuckerman and others during the 1990s [13].

**Table 1:** MAMA+ for IDU, Ukraine

Service provider networks specializing in HIV & IDUs	Psychosocial support	Harm reduction
• Early identification of HIV+ pregnant women and mothers with young children	• Psychological consultations	• Drug and alcohol rehabilitation
• Identification of pregnant women at risk of abandoning infants	• Peer network and peer support groups	• Substitution therapy
• Comprehensive antenatal and post-delivery healthcare referrals	• Legal assistance	• Other (non-specified)
• Referrals to harm-reduction services	• Material support	
• Home visits	• Child development consultations	
• IDU team comprised of team coordinator, social workers, medical professional, drug and alcohol abuse consultant, psychologist and lawyer		

MAMA+ for IDU was piloted by Health Right International in Ukraine with funding from the Open Society Institute as an extension of the *United States Agency for International Development *(USAID)-funded Prevention of Abandonment of Children Born to HIV-Positive Mothers programme (called MAMA+) offered to HIV-positive, pregnant women in Russia and Ukraine [34]. The original project set out to reduce the number of children abandoned by HIV-positive mothers through the establishment of networks of agencies and specialists to identify seropositive pregnant women and mothers. The programme identified the primary drivers of abandonment as lack of information on HIV/AIDS and prevention of vertical transmission; stigma and discrimination at medical and social institutions and by families; financial pressure and homelessness; unplanned pregnancy; and lack of social and peer support.

Thirty-five percent of MAMA+ clients were IDUs, but in the original incarnation of the intervention, their drug addiction was not taken into consideration as a risk factor requiring additional support. In order to adequately meet the needs of this substantial portion of their target group, MAMA+ conducted a six-month pilot intervention focused on providing drug-addicted women with drug and alcohol counselling, risk reduction, legal assistance and referrals [34].

The referral network was adapted to include harm reduction, drug-substitution therapy, and rehabilitation programmes. A drug and alcohol addiction consultant was hired, and new peer support groups started, focusing on the challenges created by dependence on illegal drugs. The comprehensive approach combined early identification and enrolment with home visits, and provided material, psychological and legal support (Table 1). Within six months of launching the project, 25 HIV-positive IDU pregnant women and new mothers were benefiting from services, in addition to 27 children and 19 other family members.

### TASINTA for children of sex workers

We found information on 18 organizations providing care for the children of sex workers in Bangladesh, Cote d'Ivoire, Kenya, India, Nepal, Vietnam and Zambia. The information available on the programmes was largely gleaned from Internet searching and correspondence and phone interviews with programme implementers. It is, therefore, limited in terms of programmatic detail, information about the population served, and effectiveness or long-term impact.

The interventions we found tend to provide multi-faceted assistance to mothers and children across several categories, providing children with educational opportunities and a safe place to play, study, or sleep when their mothers are working. Likewise, the same programmes provide vocational training and alternative income-generation opportunities to mothers who want to leave sex work or reduce the number of clients they need to entertain in order to provide for their families. Other types of assistance provided include peer support, nutrition, housing and healthcare.

TASINTA (We Have Changed), started in Zambia in the 1990s, is the programme for which we were able togather the most comprehensive information [personal communication, Nkandu Luo]. TASINTA started as a programme to help sex workers protect themselves from HIV, but input from the women themselves made it clear that a more broadly based, family-centred approach was necessary. A list of TASINTA's services to FSWs and their children is provided in Table 2.

**Table 2:** TASINTA (We Have Changed), Zambia

Day care	Residential care	Rehabilitation & vocational training for mothers	Education & vocational training for children	Other services
• After-school drop-in centre	• Partnership with two institutions (Kasisi Orphanage and Hope House) to provide residential care and schooling for orphans	• Drop-in centre where mothers can learn alternative skills	• Assistance with school Fees	• Help women rent homes
		• Grants for small business start up	• After-school drop-in centre	• Reunite women with children living with extended family
		• Sponsorship of higher education courses for women with secondary school education	• School-age orphans attend boarding school at Hope House	• Partner with police and government to reduce exploitation and recruit women into programme
		• Programme participants become trainers and employees		

TASINTA's partnership with residential care facilities to serve as a boarding school for children whose mothers have died may at first seem antithetical to the family-centred care model. However, it appears that TASINTA is redefining family beyond the bounds of biological relationships in the best interests of the child to include what Richter calls "long-term, mutually supportive relationships" [35].

After experimenting with reuniting orphans with extended family, TASINTA found that it was no longer able to monitor the care and safety of children and faced a situation where family members were selling the children into prostitution. Programme managers and clients working for the organization found themselves, not infrequently, searching for children and rescuing them: hence, the decision to place them in a residential environment they knew to be safe and where the children can remain close to adults they know and trust.

## Conclusions

### Methodological and ethical challenges

In order to understand the vulnerabilities faced by the families of drug users and sex workers and provide interventions designed specifically to mitigate risks and fulfill needs, identification of individuals or communities and analysis of their specific situation are necessary first steps. Yet conducting research among and even targeting the vulnerabilities faced by sex workers and drug users and their children is a methodologically and ethically challenging undertaking. Any attempt to document their needs or provide them with interventions must take care not to expose or further compromise fragile families frequently existing on the fringes of the law.

Because drug use and sex work are often illegal, those who engage in these activities are frequently referred to as "hidden" or "invisible" populations. While methodologies have been developed to reduce sampling bias, it is nearly impossible to obtain a truly random sample of such populations [5]. The very act of identifying families can also increase their vulnerability.

As noted by Family Health International, the usefulness of knowing the magnitude of vulnerable populations does not outweigh the guiding principle of public health to "do no harm" [36]: "The danger of a backlash exists not only at the individual but also at the population level, through the mere publication of information about the existence and size of a sub-population. Where there is a real possibility ... leading to harm ... it may be better to drop the whole exercise." In the case of sex workers and drug users and their children, caution must prevail in order to avoid the forced removal of children from parents, imprisonment or worse.

A case in point is the situation facing the families of drug users and sex workers in Vietnam. MOLISA's clear objective to highlight the needs of this hidden subset of extremely vulnerable children in the National Plan of Action for Children Affected by AIDS (NPA) (mentioned earlier) illustrates the complexity and possible danger of documentation and heightened attention. Despite what seem to be the good intentions to direct services to MARPs and their families, the NPA also notes contradictions between public health policy and a legal system that can increase vulnerability [10].

Identification of children whose parents use illegal drugs or sell sex may land parents in rehabilitation centres or prison, effectively leaving their dependent children to be incarcerated with their parents or placed in protection centres. These institutions are often impersonal, providing little in the way of care, and may not separate juvenile inmates from adults or offer HIV-prevention education or harm-reduction services. They may thus perpetuate the cycle of vulnerability [10,37]

The situation in Vietnam is an extreme but not anomalous example of the tension that can exist between drawing attention to vulnerable families in order to provide services and advocacy, and pushing an invisible population into a spotlight from which they have long shied away. Documenting the illegal behaviours of parents can lead to scrutiny from child welfare advocates and law enforcement, and indirectly lead to forced separation of children and parents. While such separation may reduce the immediate risks faced by an abused or neglected child, it can also do collateral damage to already fragile, but otherwise positive, family situations, leading to depression and self-blame on the part of the parent, causing distress among children and potentially jeopardizing child-parent attachment [13].

### Programme documentation and evaluation

None of the interventions we found in lower resource countries have been evaluated for short-term effectiveness or longer-term impact. Indeed, the peer-reviewed and grey literature focused on the children of drug users and sex workers is silent on many issues of critical importance for reducing their vulnerabilities, including:

◆ Strategies for accessing these often hidden, hard-to-reach families, in particular children

◆ The type of interventions that are most effective

◆ Strategies for designing, implementing and scaling up interventions for children of parents whose behaviour is illegal and perceived to be immoral in many countries.

Responding to these critical challenges would facilitate more accurate targeting of interventions toward families in need. And building an empirical evidence base of what interventions work in varying contexts would allow programme planners and implementers to be more thoughtful in choosing interventions. The establishment and enforcement of global guidance on norms and country-specific regulations that acknowledge the needs of the families engaged in illegal or "immoral" activities is essential. Before we start duplicating and scaling up any identified promising strategies, we need to document and evaluate extant programmes providing assistance to the families of drug users and sex workers, while tailoring new programmes to the needs and conditions of specific contexts.

Research from the United States and Europe is a useful place to start, but we must take up the challenge to find and (when they cannot be found) develop strategies that help to strengthen fragile families [38]. The net results of the findings from this review, the United Nations Children's Fund (UNICEF) Framework for the Protection, Care and Support of Orphans and Vulnerable Children Living in a World with HIV and AIDS [39], and the Joint Learning Initiative on Children and AIDS [40] highlight some core approaches backed by evidence, including among low- and middle-income countries.

These include integrated interventions for families and communities similar to those already being implemented by MAMA+ for IDU and TASINTA:

◆ Strengthening family caring capacity through home visitation and peer support for vulnerable parents to provide mental health support, parenting skills coaching, and monitoring of child welfare

◆ Early childhood development programmes for children, educational assistance, crèches and drop-in centres

◆ Economic strengthening and job skills training projects.

Understanding the specific context in which drug use or transactional sex interacts with a parent's ability to take care of a child is of critical importance. However, we must carefully weigh competing risks and benefits when generalizing about vulnerability, need and optimal family-centred practices. The environment in which these children live can increase vulnerability, but removing children may also mean separating them from parents who they love, and who love them and are doing their best.

In a number of ways, this literature review has generated as many questions as it has answered. We have synthesized research on the vulnerabilities faced by children of drug users and sex workers and documented two family-centred interventions being implemented in Ukraine and Zambia. But much remains to be done as we work toward implementing the UN Convention on the Rights of the Child for the children of highly vulnerable, socially marginalized parents around the world.

## Competing interests

The authors declare they have no competing interests.

## Authors' contributions

The paper was written by JB, and GB designed the literature search protocol, carried out research, and drafted and revised the manuscript. JC and JLS participated in drafting and revising the manuscript. MIB, MO, MB and DF designed the literature search protocol, carried out research, and contributed to the written content. All authors read and approved the final manuscript.
